# Use of screw locking elements improves radiological and biomechanical results of femoral osteotomies

**DOI:** 10.1186/1471-2474-15-387

**Published:** 2014-11-21

**Authors:** Gerardo L Garcés, Oliver Rodríguez, Enrique Rodríguez Grau-Bassas, Syra Roiz, Alejandro Yánez, Alberto Cuadrado

**Affiliations:** Hospital Perpetuo Socorro and Department of Medical and Surgical Sciences, Las Palmas de Gran Canaria University, c/ León y Castillo 407, 35007 Las Palmas de Gran Canaria, Spain; Hospital Veterinario Universitario, Faculty of Veterinary Medicine, Campus of Arucas, Las Palmas de Gran Canaria University, Las Palmas de Gran Canaria, Spain; Department of Comparative and Animal Medicine, Faculty of Veterinary Medicine, Campus of Arucas, Las Palmas de Gran Canaria University, Las Palmas de Gran Canaria, Spain; Department of Mechanical Engineering, Biomechanical Laboratory, Engineering Departmental Building, Campus de Tafira Baja, Las Palmas de Gran Canaria University, Las Palmas de Gran Canaria, Spain

**Keywords:** Osteotomy, Fracture fixation, Screw locking elements, Dynamic compression plate

## Abstract

**Background:**

Dynamic compression plate (DCP) constructs provide inadequate fixation in cases of poor bone quality and early weight-bearing. Screw locking elements (SLE) are flat locking nuts placed at the end of the screw to prevent screw stripping from the bone, improving fixation stability. The purpose of this work was to compare biomechanical and radiological evaluations of femoral ovine osteotomies fixed using DCP constructs with and without SLE.

**Method:**

A dyaphyseal femoral osteotomy was performed in sixteen adult sheep and fixed with a DCP and cortical screws. Half of the animals were operated on with a SLE on each side of the osteotomy and the rest without the addition of SLE. Four animals of each group were euthanized after 8 weeks, and the remaining after 16 weeks. Both femora of each animal were radiographed and mechanically tested in torsion.

**Results:**

Radiologically femoral malalignment or screw loosening was observed in six out of the eight animals operated on without SLE. In contrast, all animals subjected to the operation with SLE showed complete radiological consolidation of the osteotomy. Seven of these eight animals showed normal femoral alignment and no osteosynthesis failure. Stiffness of the bones fixed with SLE was among 145% and 177% the value of their contralateral non-operated femurs (all animals of this group showed greater stiffness on the operated bone than its contralateral non-operated femur). However, stiffness of the bones operated on without SLE was among 58% and 87% the value of the stiffness of their contralateral non-operated bone (all animals of this group showed greater stiffness on the non-operated bone than the osteotomized ones).

**Conclusions:**

Use of SLE avoided loosening of the system and stimulated stronger osteotomy consolidation. Clinical application of this improved system may thus be a feasible and cost-effective alternative to other more rigid and expensive bone fixation techniques.

**Electronic supplementary material:**

The online version of this article (doi:10.1186/1471-2474-15-387) contains supplementary material, which is available to authorized users.

## Background

The open reduction and internal fixation of fractures is one of the common used options to treat instable fractures. The stability of this fixation is mainly dependent on anchorage of the screw in the bone. During initial stabilization, poor bone quality, such as that observed in osteoporosis, disuse osteopenia or hidden fissure lines, screw overtightening and excessive mechanical demand may cause inadequate screw anchorage, leading to less stable bone fixation and subsequent osteosynthesis failure. The result of this may be residual pain, delayed union or nonunion and bone misalignment.

The development of locking plates (LP) has provided a suitable alternative to standard compression plates. LP contain threaded screw holes that support rigid engagement of threaded locking screw heads with the plate. The resulting locking plate constructs provide considerable fixation strength via fixed-angle stabilization, rather than plate-to-bone compression required with conventional non-locked plating constructs. However, earlier experimental data have suggested that the overall construct stiffness of LP systems is increased[1–4]. Stiffness of locked plating constructs can suppress interfragmentary motion to a level that is insufficient to reliably promote secondary fracture healing by callus formation[5]. Ideal stiffness may be significantly less than that achieved with these locked constructs, and overly stiff constructs may lead to impaired fracture healing and stress concentration at the ends of the plate[4, 6]. The ideal construct necessitates maintenance of screw purchase and fracture reduction until healing is complete while allowing sufficient, but not excessive, fracture micromotion[2, 4, 7].

The plate-screw rigid interface causes uneven stress distribution, whereby stress risers cause bone fracture at the end screw[6] and stress shielding under the plate can lead to bone resorption[2]. From a clinical viewpoint, the stiffness of the standard locked plating constructs and complications observed with their use are issues of increasing concern[8, 9]. Moreover the high price of this technology presents an important restriction for many surgeons. Developing countries and those with a small budget health care system are rarely able to employ these techniques due to affordability constraints[10–12].

To improve the fixation stability of conventional unlocked systems, Yanez *et* al.[13] proposed the use of screw locking nuts, placed at the ends of the screw shafts, which have been denoted 'screw locking elements' (SLE). These can be easily applied through a specially manufactured device[14] and their use provide to DCP constructs similar fixation strength that LCP constructs, with significant less stiffness[15].

In view of the evident mechanical benefits of SLE *ex vivo*[13, 15], we further aimed to validate these results *in vivo*. In the current investigation, we have performed biomechanical and radiological comparative evaluation of ovine femoral osteotomies fixed with DCP and standard screws with and without SLE.

## Methods

Sixteen skeletally mature, healthy female Merino-mix sheep (2.5 to 3.5 years old with mean ± SD weight of 63 ± 8 kg) were divided into two groups of eight animals each. This study was carried out according to the policies and principles established by the Spanish Health Authorities in their Guide for Care and Use of Laboratory Animals as well as the European Animal Welfare Guidelines (86/609/EEC), and approved by the local legal representative (University of Las Palmas de Gran Canaria Ethics Committee for Animal Welfare: registration number 007/2010 CEBA ULPGC).

### Animal Models and Surgical Procedures

Under general anesthesia with isoflurane, the right hind leg was sterilely draped and a 12 to 15 cm long lateral incision made over the femur. After longitudinal incision of the fasciae latae, the vastus lateralis was lifted from the femur coagulating the small penetrating arteries. A standard narrow 4.5 mm stainless steel DCP plate (Zimmer Inc, Warsaw, Indiana) with six or eight holes was bent to fit the lateral face of the diaphysis of the bone at an approximately equidistant position between the greater trochanter and lateral femoral condyle. Eight animals were randomly operated on and fixed with a plate of 6 screws (group 6 s), and the remaining eight subjected to osteotomy with a plate of 8 screws (group 8 s).

**Figure 1:**
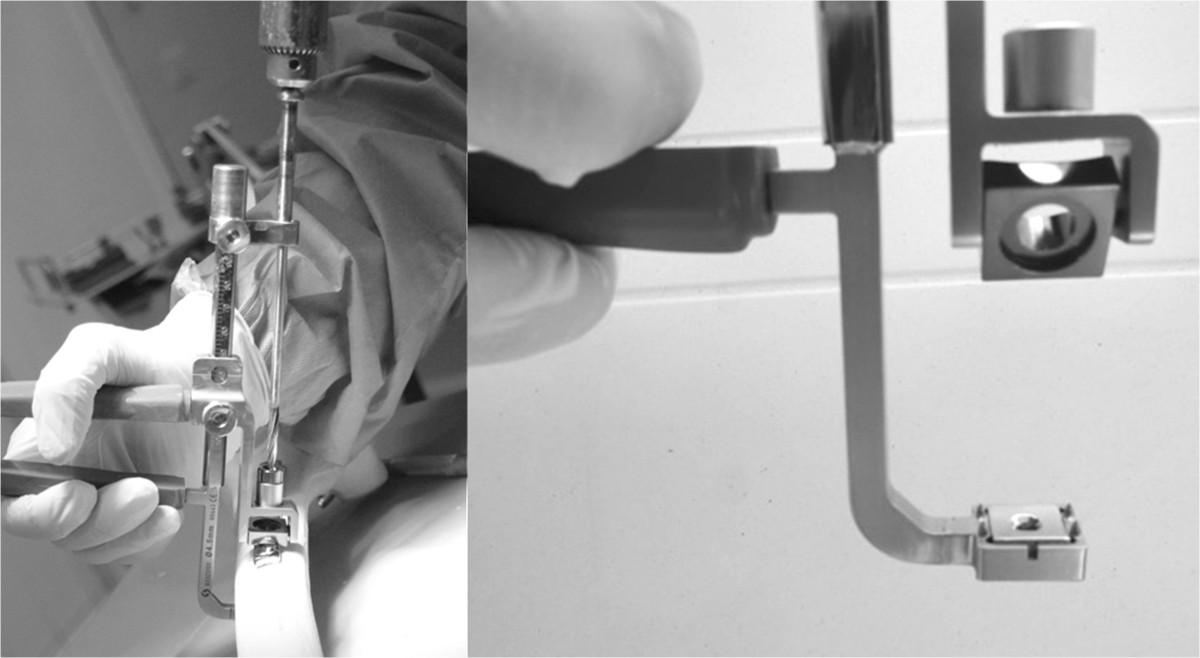
**Device used to place the SLE on the screw beyond the far cortical.**

Tapping screws of 4.5 mm were used, their length protruding 2 mm beyond the far cortical. A expert surgeon in osteosynthesis applied manual torque, similar to the normal clinical setting, to fit the screws. In four animals of each group, a SLE (Surgival, Valencia, Spain) was placed on the tip of the screw at position 2 from the osteotomy on each side. The SLE, measuring 12×12×4 mm, was manufactured from the same surgical steel as the screws. These elements have a central threaded 4.5 mm in diameter hole to fit the screw. A specifically designed device[13] was used to place the SLE in the appropriate position (Figure 1). A 4.5 mm screw hole was drilled to permit smooth screw insertion into the SLE. For the rest of the screws, a drill bit of 3.2 mm was used. Manual torque was applied to fit the screw and SLE until sufficient resistance was encountered in a similar way to that used for screws without SLE.

After placing the plates and screws, a transverse osteotomy was made at the middle point of the plate using a Gigli saw 1.5 mm in diameter. No external immobilization was utilized, and animals were allowed to move and walk freely following the operation, with food and drink provided *ad libitum*. One gram intravenous Cefuroxime was used as antibiotic prophylaxis, and administered both pre and 24 hours postoperatively. Analgesia with Bupremorfine (5 micrograms/Kg twice a day) and Flunixin (1 mg/Kg once a day) was applied for the first week. A qualified veterinarian was exclusively dedicated to animal care during the whole project. Radiographs of the operated limb were obtained immediately after the operation and every four weeks thereafter.

Animals of group 8 s were sacrificed 8 weeks after the operation and animals of group 6 s after 16 weeks, using Thiopental overdose. Both femurs were harvested from each animal. Radiographic images of bones were obtained in the antero-posterior and lateral positions, and hardware removed from the femurs subjected to operation. Criteria for radiological assessment were as follows: a) number of periosteal bridges in the antero-posterior and lateral views (range 0–4), b) femoral alignment in comparison with the contralateral femur, c) loosening of screws in relation to immediate postoperative radiography.

After hardware removal, mechanical testing was performed on both unoperated and osteotomized femora. The proximal and distal ends of the bones were rigidly embedded in mounting fixtures with the use of bone cement and the bone suspended in a torsional testing system perfectly aligned (Microtest, Madrid, Spain). To minimize alignment artifacts, the proximal fixture was attached to a universal joint that permitted rotations around the anteroposterior and mediolateral axes but prevented rotation around the diaphyseal axis[3]. Rotation around the femur shaft axis was applied at a rate of 10° per minute under an axial preload of 20 N. Torsional stiffness was calculated from the linear slope of torsion versus the rotation curve.

For statistical analysis, data are presented as mean values and standard deviation of the whole SLE-fixed group and the non SLE-fixed group as well as mean values of each group. Statistical differences between the operated and their non-operated contralateral bones were assessed with the Wilcoxon test at a level of significance of p < 0.05. Differences between osteotomized bones with and without SLE were assessed with a T-test for unpaired samples.

## Results

Radiographs obtained immediately after surgery showed normal alineation of bones with screws and plates, indicating that SLE was appropriately placed in all the animals. Mobility of animals was fairly restricted for the first week after the operation, especially those subjected to the operation without SLE. Animals were fed by the veterinarian until they were able to feed independently.

### Mechanical testing

The results of individual mechanical testing are showed in Table 1. After 8 weeks, stiffness of the femurs operated on without SLE showed between 77% and 86% the value of the stiffness of their contralateral non-operated femurs. Femora subjected to osteotomy and fixed with SLE showed between 153% and 177% the value of the stiffness of their contralateral non-operated femur. Mean value of the stiffness of the SLE osteotomized bones was significatively greater than that of non SLE osteotomized bones (2.12 ± 0.32 vs 1.32 ± 0.23, p = 0.032).

**Table 1:** **Torsional stiffness (Nm/deg) of all the animal's femora**

		Sheep number	Osteotomized bone	Non operated bone	Difference	Percentual difference
**Group 6 s**	**DCP + 6 s**	**1**	1.2450	1.8961	0.6511	65.7
		**2**	1.9512	3.3641	1.4129	58.0
		**3**	1.6870	2.4488	0.7618	68.9
		**4**	0.9359	1.5825	0.6466	59.1
		Mean ± SD	1.45 ± 0.45^a^	2.32 ± 0.78		
	**DCP + 6 s + 2SLE**	**1**	2.6340	1.8145	−0.8195	145.2
		**2**	2.8822	1.8644	−1.0178	154.6
		**3**	2.9860	2.1230	−0.8630	140.7
		**4**	1.7810	1.1800	−0.6010	150.9
		Mean ± SD	2.57 ± 0.55*^b^	1.75 ± 0.40		
**Group 8 s**	**DCP + 8 s**	**1**	1.4410	1.6611	0.2201	86.8
		**2**	1.0790	1.3860	0.3070	77.9
		**3**	1.6130	1.8819	0.2689	85.7
		**4**	1.2360	1.4949	0.2589	82.7
		Mean ± SD	1.34 ± 0.23^c^	1.61 ± 0.22		
	**DCP + 8 s + 2SLE**	**1**	2.2540	1.2710	−0.9830	177.3
		**2**	1.8401	1.2000	−0.6401	153.3
		**3**	1.8880	1.0630	−0.8250	177.6
		**4**	2.5130	1.5670	−0.9460	160.4
		Mean ± SD	2.12 ± 0.32**^d^	1.28 ± 0.21		

Group DCP + 6 s: plate of 6 screws without SLE euthanized after 16 weeks; Group DCP + 6 s + 2SLE: plate of 6 screws with 2 SLE euthanized after 16 weeks; Group DCP + 8 s: plate of 8 screws without SLE euthanized after 8 weeks; Group DCP + 8 s + 2SLE: plate of 8 screws with 2 SLE euthanized after 8 weeks.

*p = 0.032 when comparing values of osteotomized bones of DCP + 6 s + 2SLE group with values of osteotomized bones of DCP + 6 s group (T test for unpaired samples).

**p = 0.002 when comparing values of osteotomized bones of DCP + 8 s + 2SLE group with values of osteotomized bones of DCP + 8 s group (T test for unpaired samples).

^a^p = 0.018 when comparing values of osteotomized bones with their contralateral non operated bones of DCP + 6 s group (T test for paired samples).

^b^p = 0.002 when comparing values of osteotomized bones with their contralateral non operated bones of DCP + 6 s + 2SLE group (T test for paired samples).

^c^p = 0.001 when comparing values of osteotomized bones with their contralateral non operated bones of DCP + 8 s group (T test for paired samples).

^d^p = 0.002 when comparing values of osteotomized bones with their contralateral non operated bones of DCP + 8 s + 2SLE group (T test for paired samples).

After 16 weeks, stiffness of the bones operated on without SLE showed between 58% and 68% the value of the stiffness of their contralateral non-operated femur. Femora subjected to osteotomy and fixed with SLE showed between 145% and 155% the value of the stiffness of their contralateral non-operated femur. Mean value of the stiffness of the SLE osteotomized bones was significatively greater than that of non SLE osteotomized bones (2.57 ± 0.55 vs 1.45 ± 0.45, p = 0.007).

All animals showed greater stiffness of the SLE-stabilized bone than its contralateral non-operated bone (mean value was 2,34 ± 0,47 *vs* 1,51 ± 0,38, p = 0.012). All animals showed lesser stiffness of the non-SLE fixed femur than its contralateral non-operated bone (mean value was 1,39 ± 0,33 *vs* 1,96 ± 0,65, p = 0.012) (Table 2).

**Table 2:** **Mean ± SD values of torsional stiffness (Nm/deg) of SLE and non-SLE operated on groups**

		n	Mean ± SD	95% confidence interval
**Non-SLE group**	Osteotomized	8	1.39 ± 0.33*	1.19 - 1.62
	Non-operated	8	1.96 ± 0.65	1.59 - 2.45
**SLE group**	Osteotomized	8	2.34 ± 0.47*	2.03 - 2.65
	Non-operated	8	1.51 ± 0.38	1.26 - 1.76

*p = 0.012 when comparing osteotomized with their contralateral non-operated bones (Wilcoxon test).

### Radiological findings

**Figure 2:**
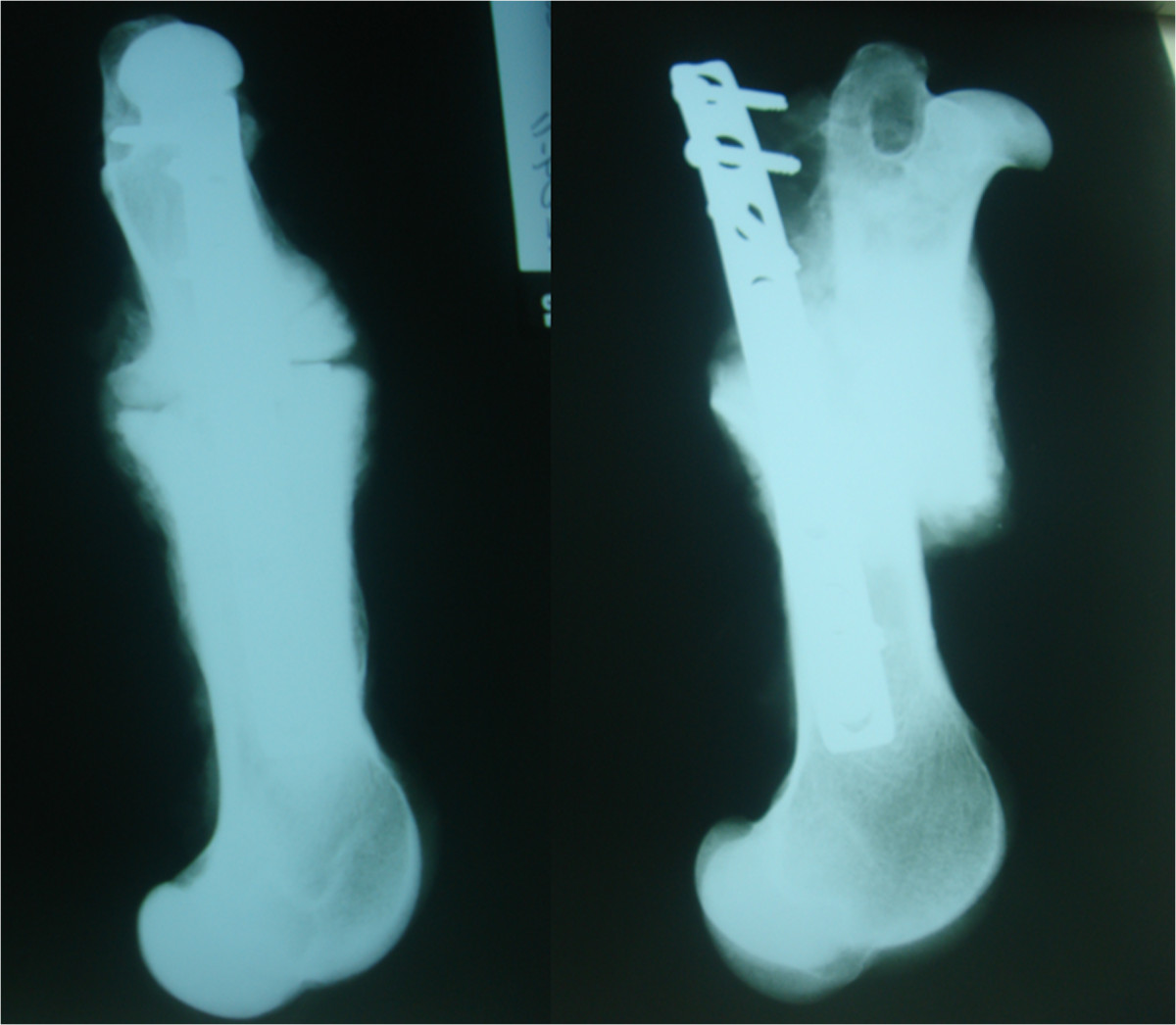
**Two examples of complications in animals operated on without SLE after 8 weeks: absence of callus bridging (left) and loosening of all proximal screws (right).**

**Figure 3:**
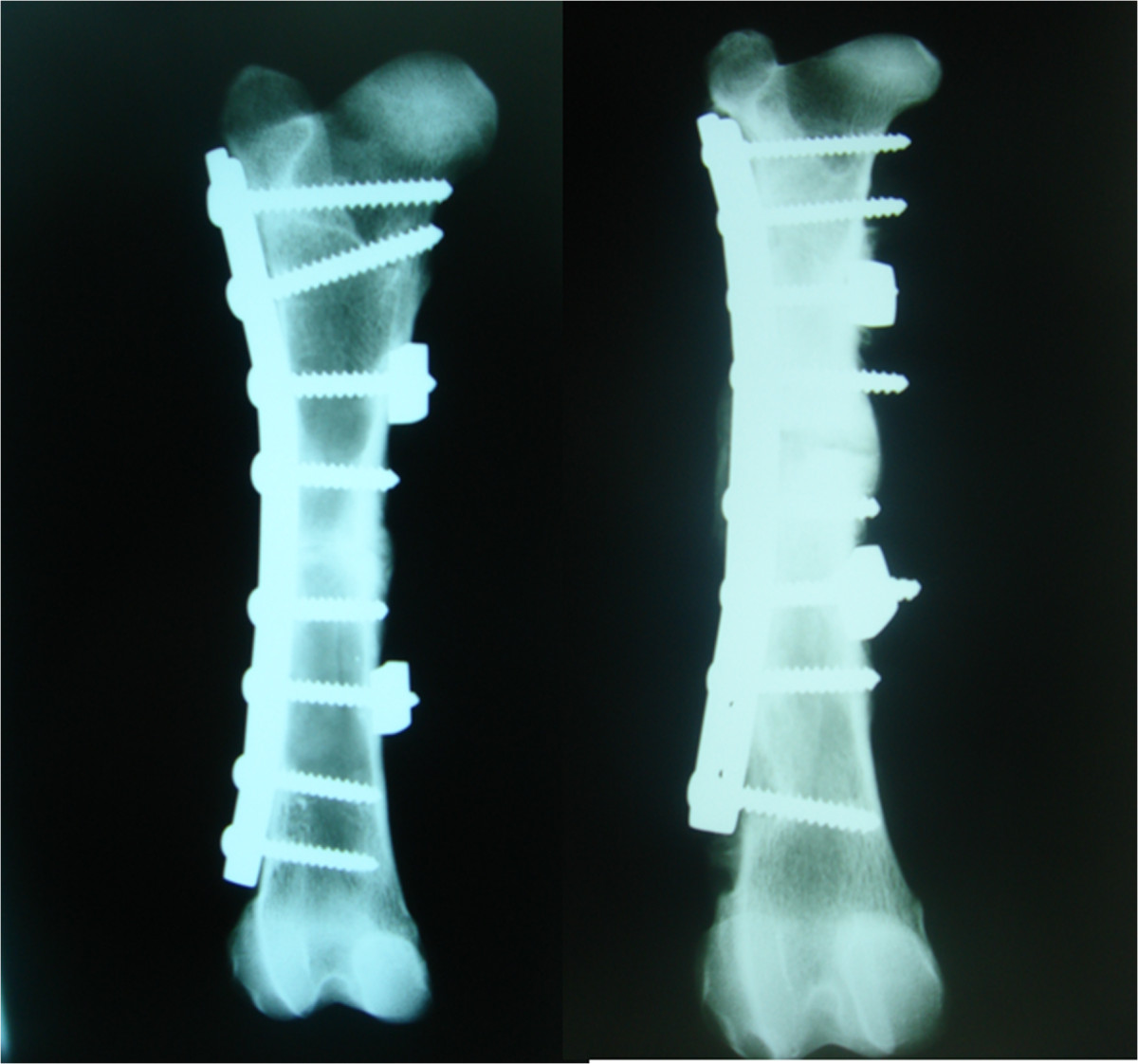
**Two examples of bones operated on with SLE after 8 weeks.** Both of them show complete consolidation of the osteotomy and normal alineation.

**Figure 4:**
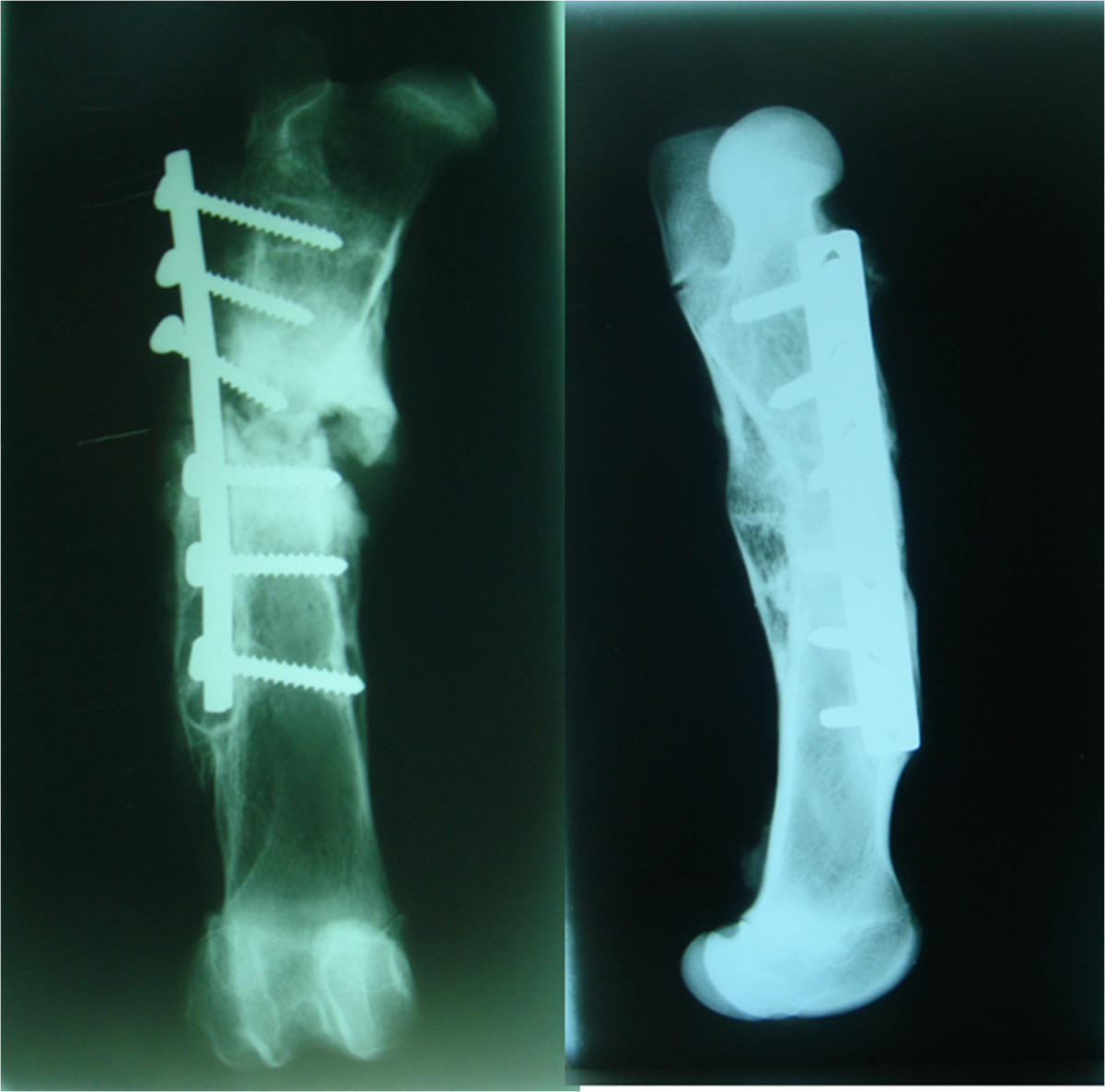
**Two examples of complications in animals operated on without SLE after 16 weeks: absence of consolidation and loosening of all proximal screws on the left and approximately 30° of femoral recurvatum on the right.**

**Figure 5:**
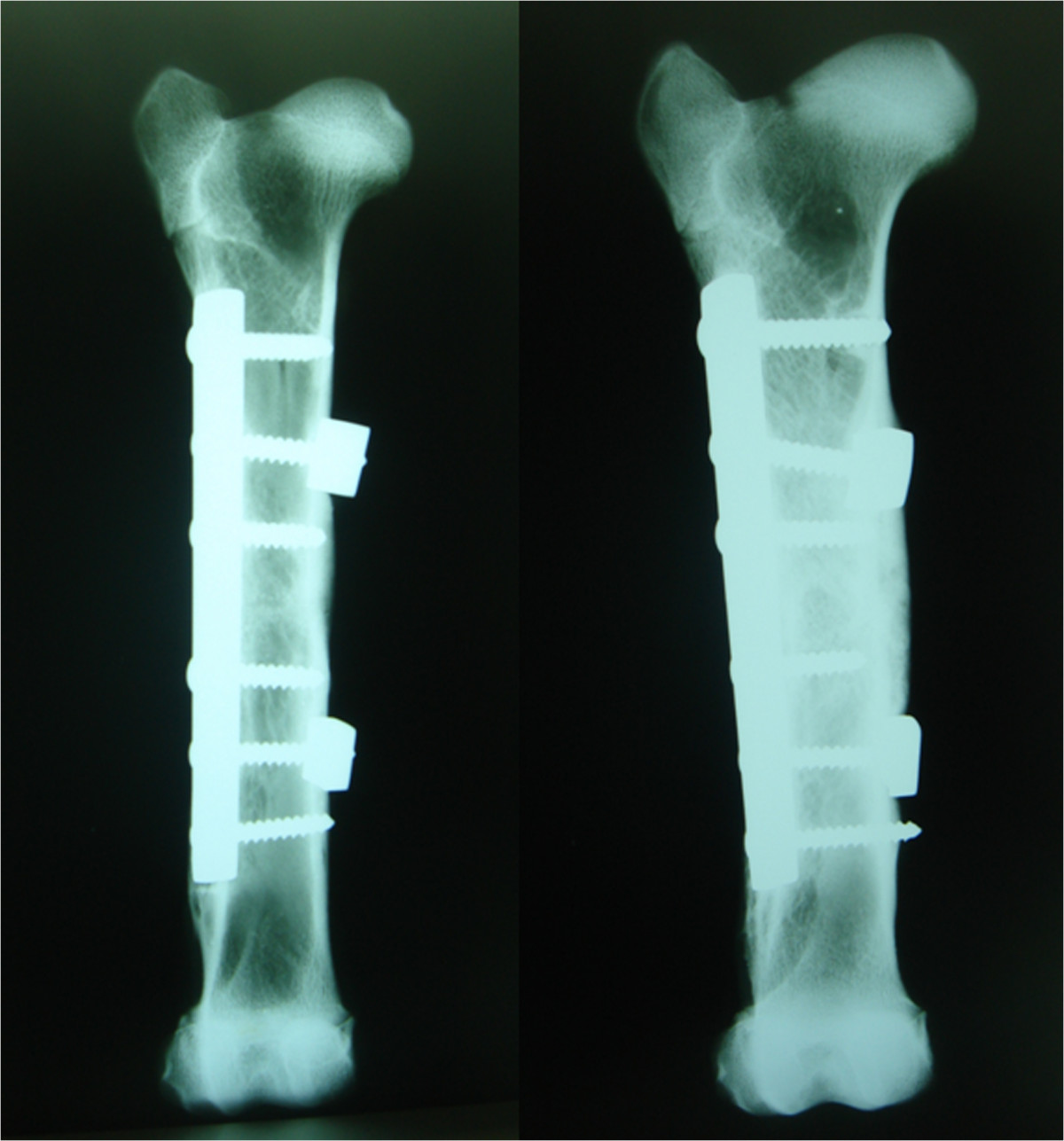
**Two examples of bones operated on with SLE after 16 weeks.** Both of them show complete consolidation of the osteotomy and normal alineation.

Radiological results are summarized in Table 3. After 8 weeks, only one of the four animals subjected to the operation without SLE showed osteotomy consolidation with four bridged corticals, normal alignment and no osteosynthesis failure. Another animal showed complete consolidation with a varus deviation of 30°. A third animal showed only one bridged cortical with signs of delayed healing, normal alignment, and one loose screw. The fourth animal exhibited complete absence of consolidation with loosening of all proximal screws (Figure 2). In contrast, all the animals operated on with SLE showed complete consolidation and normal alignment of bones (Figure 3). One of the animals in this group displayed loosening of a screw without SLE. However, consolidation and alignment remained normal in this case. No failure of osteosynthesis was observed in the rest of the animals.After 16 weeks, all the animals operated on without SLE showed recurvatum greater than 20° (Figure 4), with one case recorded as 30° plus 30° of varus. This specimen displayed clear signs of pseudoarthrosis and loosening of all proximal screws (Figure 4). The other three animals showed consolidation of osteotomy. One of these animals presented a loosened screw. All four animals in the SLE group showed complete consolidation of osteotomy, and three displayed normal alignment of the bone (Figure 5). One case showed recurvatum of 15° and varus of 10°. No screw loosening was evident in this group of animals.

**Table 3:** **Radiological findings**

		Sheep number	Cortical bridging	Femoral alignment	Osteosynthesis failure
**Group 6 s**	**DCP + 6 s**	**1**	4	15° recurvatum	1 loose screw
		**2**	4	30° recurvatum	No
		**3**	4	30° recurvatum	No
		**4**	0	30° recurvatum and 30° varus	Loosening of all proximal screws
	**DCP + 6 s + 2SLE**	**1**	4	Normal	No
		**2**	4	Normal	No
		**3**	4	Normal	No
		**4**	4	15° recurvatum and 10° valgus	No
**Group 8 s**	**DCP + 8 s**	**1**	1	Normal	1 loose screw
		**2**	0	Complete disalignment	Loosening of all proximal screws
		**3**	4	Normal	No
		**4**	4	30° varus	Plate bending
	**DCP + 8 s + 2SLE**	**1**		Normal	No
		**2**		Normal	No
		**3**		Normal	1 loose screw
		**4**		Normal	No

Group DCP + 6 s: plate of 6 screws without SLE euthanized after 16 weeks; Group DCP + 6 s + 2SLE: plate of 6 screws with 2 SLE euthanized after 16 weeks; Group DCP + 8 s: plate of 8 screws without SLE euthanized after 8 weeks; Group DCP + 8 s + 2SLE: plate of 8 screws with 2 SLE euthanized after 8 weeks.

## Discussion

The mechanical stability provided by DCP-unlocked screw constructs is usually sufficient to maintain fixation until fracture consolidation in normal bones. However, in cases of poor bone quality or high mechanical demand before consolidation, this fixation may be insufficient, leading to loosening of screws, and consequently, delay or nonunion of the fracture or inadequate bone alignment. The findings of the current study support this observation. While the number of experimental animals was relatively small, the majority of those subjected to osteotomy with DCP and screws alone displayed some of the above mentioned complications.

The use of SLE in our animals provided an objective benefit from both radiological and biomechanical points of view. All animals operated on with SLE showed consolidation of osteotomy after 8 weeks, with only a single subject displaying a small malalignment of the bone. Although one of these animals presented a loose screw without SLE, this had no influence on alignment or consolidation of bone, suggesting that just a single SLE provides sufficient stability to counter failure of fixation by one of the remaining screws. No radiological or postmorten signs of loosening or intolerance to SLE were observed in animals, indicative of perfect tolerance.

Our data provide biological confirmation of the *ex vivo* results obtained on surrogate osteoporotic bones by the group of Yanez[13, 15]. A single SLE placed on each side of the osteotomy provided enough stability to prevent failure of DCP with standard screw constructs. The majority of earlier studies on long bone fractures in sheep have been performed on tibiae[3, 16, 17], whereas we focused on femurs. In this work we have conclusively shown that SLE provides stability, even under conditions of high mechanical stress such as odd bones and unrestricted weight bearing following the operation.

The high failure rate of the DCP internal fixation system in repair of osteoporotic, early weight-bearing and poor-quality bone fractures has been clearly documented[18–22]. Failures of these constructs occur mainly due to screw loosening before fracture consolidation. To improve stability of fixation in cases of osteoporotic or osteopenic bones it has been developed the locking plate technology. This relies on screws threaded into the plate, minimizing the importance of bone quality for stabilization of the fixation system. In cases of anatomical reduction and interfragmentary compression, the LCP system possibly provides the optimal situation for primary bone healing. However, when there is no complete contact between fragments, secondary bone healing is necessary. This type of healing requires some interfragmentary micromovement to ensure success. In this sense, the ideal construct should be strong enough to avoid screw and plate fixation failure, but at the same time, have sufficient flexibility to facilitate secondary healing of the fracture[17, 23].

Since excessive rigidity is one of the main recognized disadvantages of locked plating technology, several investigators have attempted to resolve this problem without impairing the strength and stability provided by these systems. Botlang *et al*.[1–3] proposed the use of a strategy known as ‘far cortical locking’ (FCL). This basically involves increasing the drill diameter for the first (near) cortical bone, allowing the screw to only engage with the second (far) cortical bone. The authors reported a significant reduction in axial stiffness, along with a modest reduction in axial strength and increase in torsional and bending strength. The effect of FCL was further confirmed *in vivo* using an established ovine tibial osteotomy model[3]. Along similar lines, Gardner *et al*.[4] concluded that by replacing slots with holes in the near cortex under a locked plate, axial stiffness of the LCP could be reduced while maintaining construct stability. Far cortical locking reduces the stiffness of a locked plating construct, but requires accurate application to obtain a desired motion envelope in the near cortex. If a far cortical locking screw is in contact with the near cortex on insertion, it may reduce or prevent elastic flexion of the screw shafts, and thereby impede reduction of construct stiffness[3].

Locking a screw with a SLE beyond the far cortical allows micromovement at the plate-screw interface as well as within the cortex envelope of the screw. In this sense, a recent study has demonstrated that after 10,000 cycles, interfragmentary micromovement at the far cortical was 70% greater in DCP-8screw-2SLE constructs than the LCP-8 locked screw constructs[24]. Fixation strength of the construct is maintained, since engagement of the screw by the SLE is independent of the bone quality. While SLE avoids screw disengagement of the locked screw limiting the movement of the fracture focus, the remaining screws (without SLE) contribute to fixation of the construct.

As it has been shown *exvivo* fixation strength provided by the SLE avoids loosening and stripping of the screw, similar to LCP constructs[13]. However the excessive stiffness achieved with LCP is not observed with the use of a single SLE on each side of the osteotomy[15]. The use of SLE avoids screw disengagement from the plate independent of bone quality. In our study, a gap of nearly 2 mm was created to simulate a poorly reduced fracture. The high index of failures observed in our animals operated on without SLE suggests that the DCP-non SLE construct is a poor option to treat diaphyseal fractures of weight-bearing bones early after the operation. Conversely, results obtained from our animals subjected to osteotomy and fixed with SLE demonstrate that these locking nuts provide sufficient fixation stability to stimulate osteotomy consolidation without failing construct. To implant the SLE was necessary to strip off the muscle from the bone by around 15 mm to place them beyond the far cortical, however this had no negative effect on bone healing in our animals.

Our study has a few limitations. Firstly, the number of animals under investigation was small. However, due to the high mortality and morbidity in the not SLE operated on group we decided not to increase the number of animals. Since the average results of stiffness of osteotomized bones were quite similar at 8 and 16 weeks, both for the SLE treated group and for the not SLE operated one, we considered that time passed after the operation and number of screws used had no influence in the differences observed in our work. We think that both radiological and biomechanical findings of our work were enough to validate the reproducibility of the results[3, 25, 26]. Biomechanical tests were conducted only in torsion, and we do not know if the results would be consistent with those of mechanical tests in flexion or compression. However, torsional tests are most widely used *in vivo*, and considered sufficient to reach valid conclusions from the biomechanical point of view[2, 3, 16, 26]. Moreover, numerical quantification of fracture callus was not carried out, as it would be a more objective assessment[3]. Despite its questionable inter-observer reliability, we employed the traditional method of considering cortical bridging of at least three out of four corticals as a definition of radiological healing, as recommended by several working groups[27–30]. The torque applied to the screws, including those with SLE, was not quantified. However, similar to the normal clinical setting, this seems unnecessary, since the results were homogeneous and no adverse effects were observed around the SLE.

Use of locking nuts is not new in trauma surgery. Schuhli locking nuts (Synthes, Paoli, PA, U.S.A.) are stainless steel discs with a threaded hole in the center which, when placed between the plate and bone, act to elevate the plate from the bone and lock the screw to the plate. This creates a low-profile internal fixator which improves stability of not locked constructs[31]. However this concept has now been substituted by the locking plate technology where screws are directly locked to the plate. Use of nuts located at the free end of the screw, beyond the far cortical, has been abandoned due to the big size of the nuts and difficulties inherent to their surgical placing. With the specially designed nuts and device to implant them used in our work the surgical technique is easy and the results are reproducible. While the system developed in this study needs to be validated clinically, our preliminary results suggest that this simple and cost-effective technology is applicable in multiple clinical situations, both for humans and animals. In fact, several patients (clavicle and humeral fractures) have been treated at the first author's institution with this technology showing excellent results with no complications (unpblished results).

## Conclusions

Currently, unlocked screws and plates continue to be the most widely used method in osteosynthesis. The main concern associated with this technology is loosening of the system in cases of osteoporosis or poor bone quality and also in cases of early weight-bearing. Data from the current investigation have proved that ovine diaphyseal femoral osteotomies fixed only with DCP and screws showed a high rate of consolidation failures. In contrast, the addition of a single SLE to each side of the osteotomy fixed with the same DCP-screw system provided sufficient increase in the fixation stability to prevent failure of the construct. Despite unrestricted weight bearing of animals following the operation, no osteosynthesis failure was observed in the osteotomy group fixed with SLE. Moreover, the use of these locking nuts allowed bone healing and faster osteotomy consolidation in comparison with bones operated on without SLE.

## Authors’ original submitted files for images

Below are the links to the authors’ original submitted files for images. Authors’ original file for figure 1 Authors’ original file for figure 2 Authors’ original file for figure 3 Authors’ original file for figure 4 Authors’ original file for figure 5
