# Supplementary figures and images for: Use of screw locking elements improves radiological and biomechanical results of femoral osteotomies
Source: BMC Musculoskelet Disord. 2014 Nov 21;15:387. doi: 10.1186/1471-2474-15-387 (PMC4258045; doi:10.1186/1471-2474-15-387)

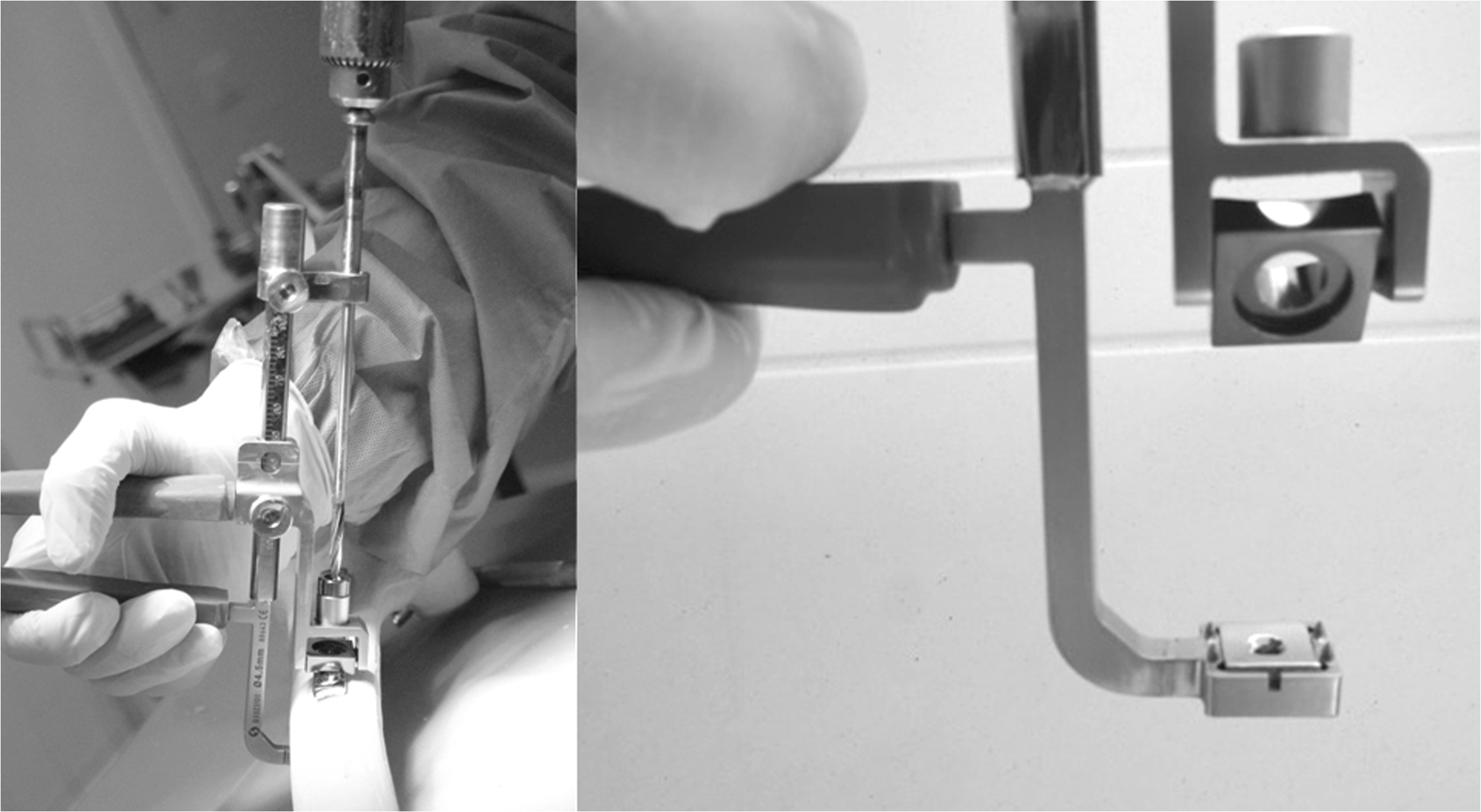

Supplement: Supplementary file 1 — Authors’ original file for figure 1 [file 12891_2013_2336_MOESM1_ESM.tif]

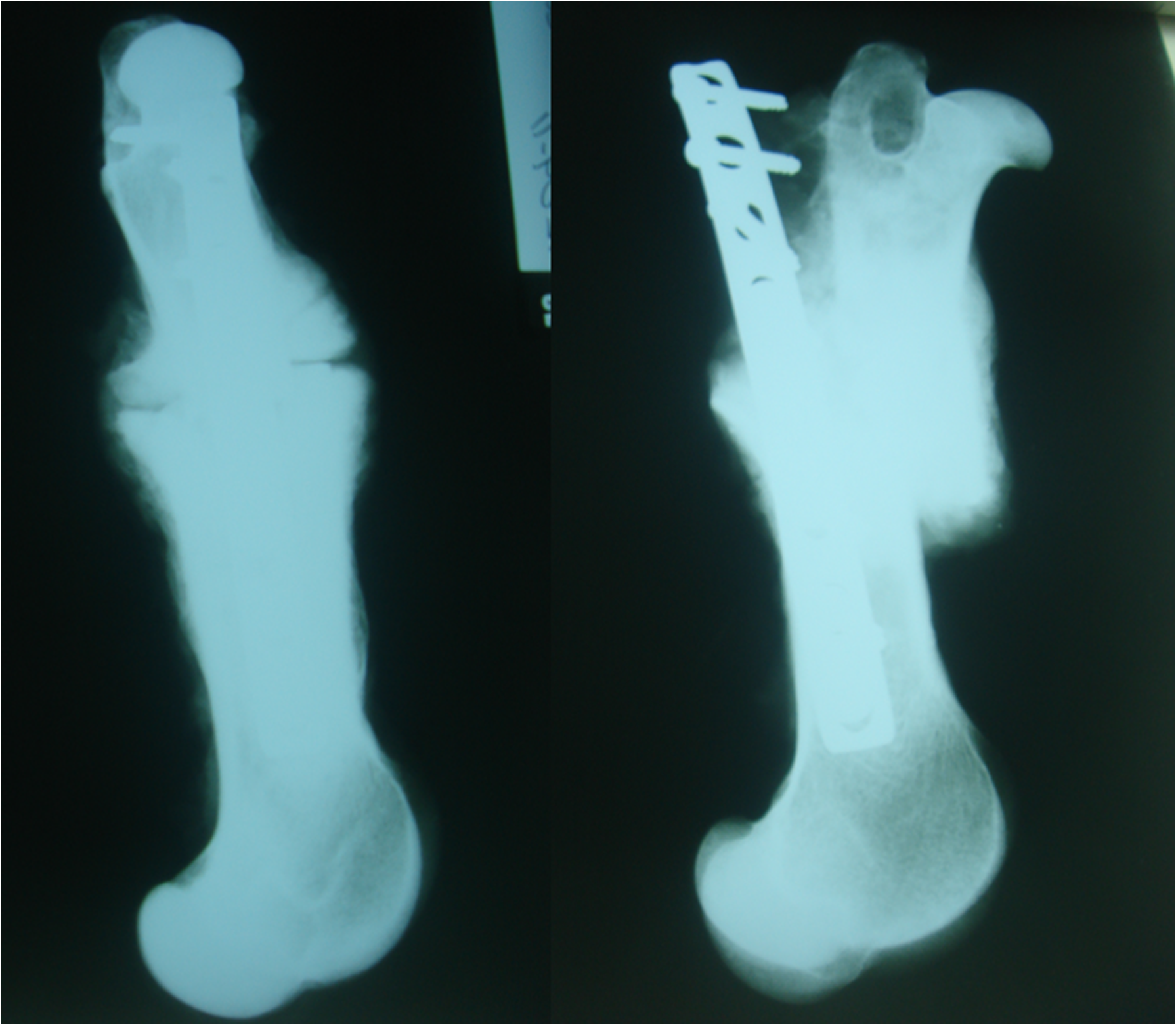

Supplement: Supplementary file 2 — Authors’ original file for figure 2 [file 12891_2013_2336_MOESM2_ESM.tif]

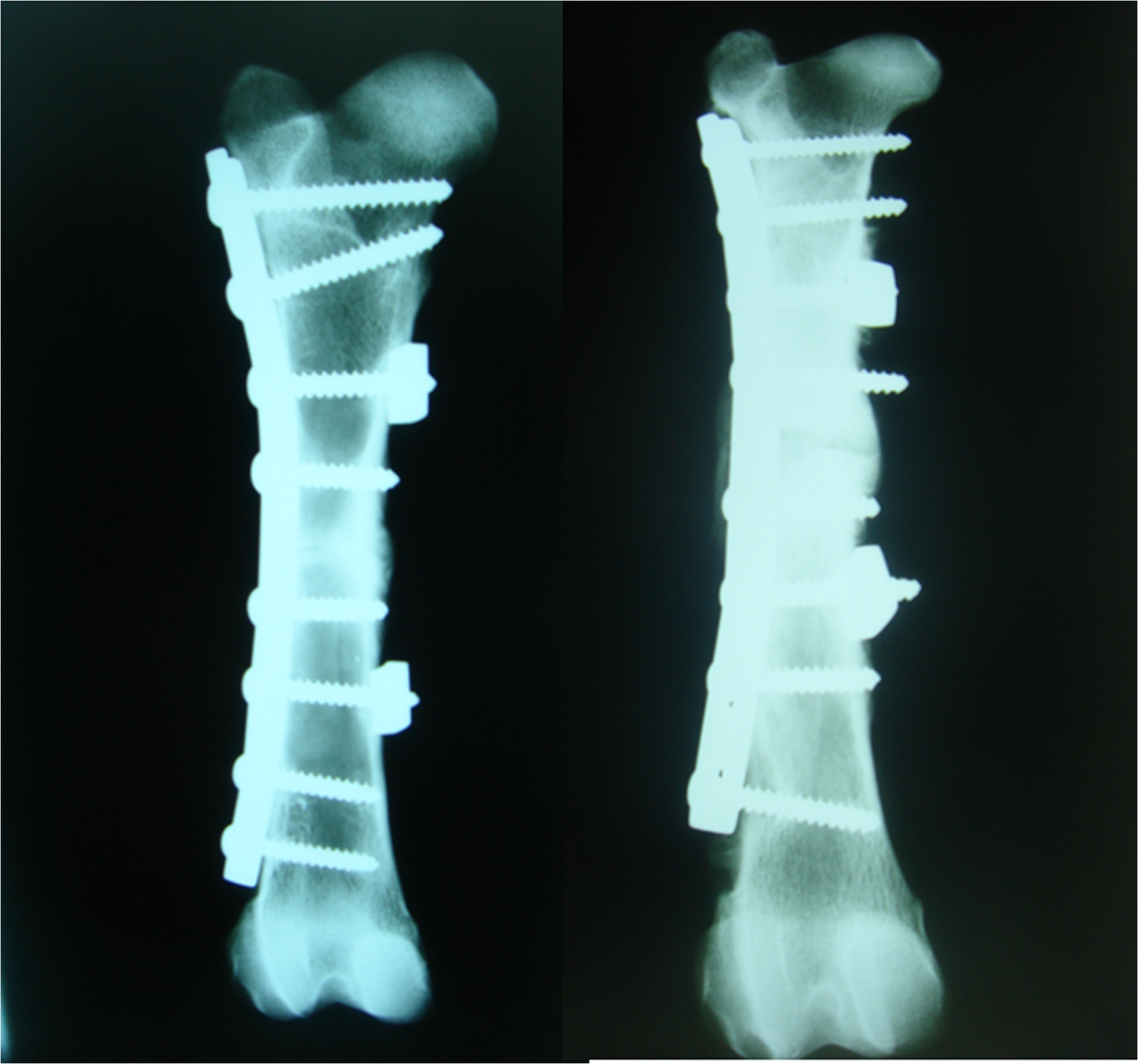

Supplement: Supplementary file 3 — Authors’ original file for figure 3 [file 12891_2013_2336_MOESM3_ESM.tif]

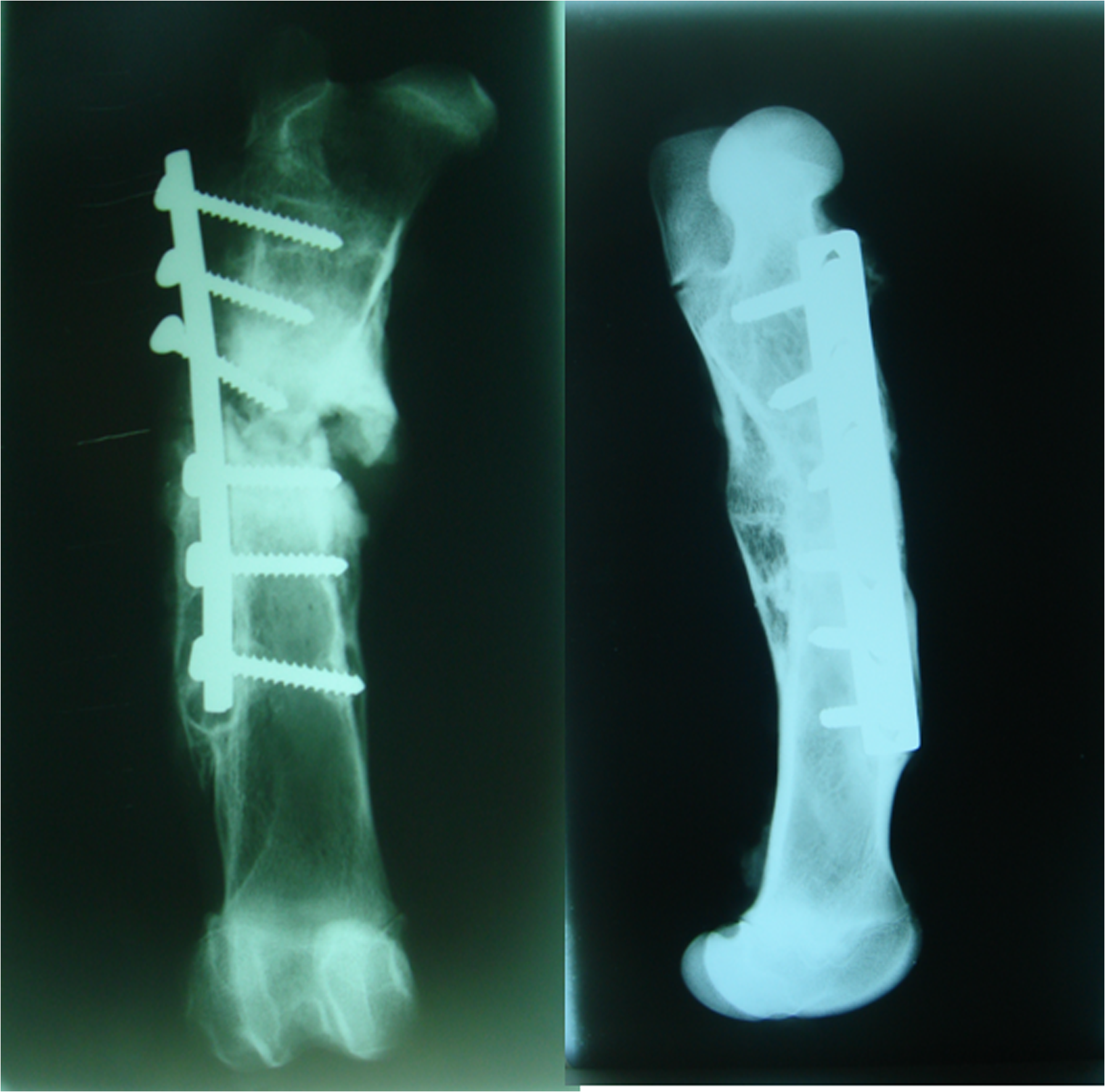

Supplement: Supplementary file 4 — Authors’ original file for figure 4 [file 12891_2013_2336_MOESM4_ESM.tif]

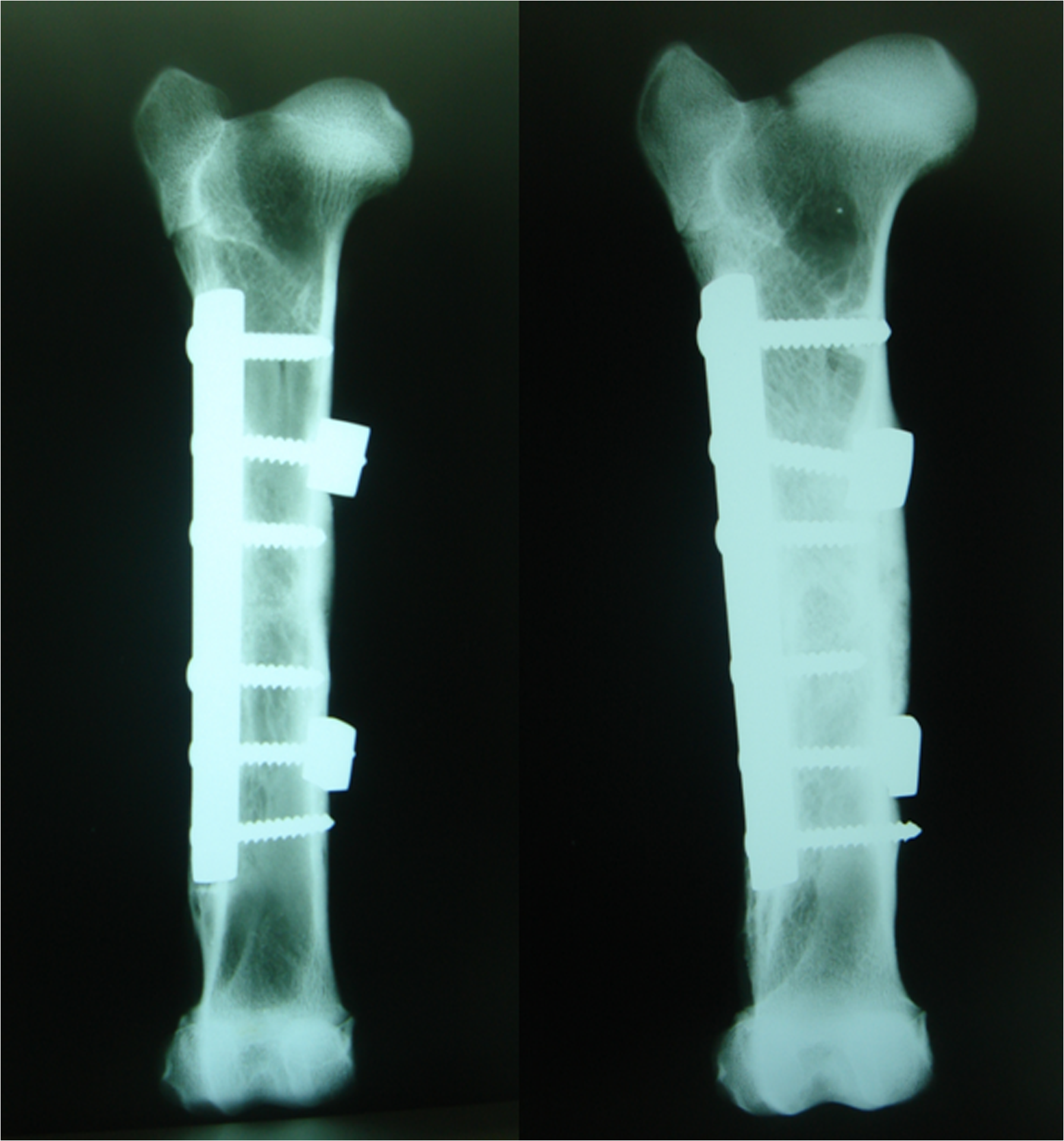

Supplement: Supplementary file 5 — Authors’ original file for figure 5 [file 12891_2013_2336_MOESM5_ESM.tif]
